# Psychological Stress Up-Regulates CD147 Expression Through Beta-Arrestin1/ERK to Promote Proliferation and Invasiveness of Glioma Cells

**DOI:** 10.3389/fonc.2020.571181

**Published:** 2020-10-15

**Authors:** Ping Wang, Zhenming Wang, Yizhi Yan, Lin Xiao, Wenxiu Tian, Meihua Qu, Aixia Meng, Fengxiang Sun, Guizhi Li, Junhong Dong

**Affiliations:** ^1^ Department of Biochemistry, School of Basic Medicine, Weifang Medical University, Weifang, China; ^2^ Department of Clinical Laboratory, Weifang City People’s Hospital, Weifang, China; ^3^ Translational Medical Center, Weifang Second People’s Hospital, Weifang, China; ^4^ Central of Translation Medicine, Zibo Central Hospital, Zibo, China

**Keywords:** glioma, psychological stress, invasion, CD147, matrix metalloproteinases

## Abstract

Psychological stress is closely related to the occurrence and prognosis of various malignant tumors, but the underlying mechanisms are not well studied. CD147 has been reported to be expressed in glioma and other malignant tumors. CD147 not only participates in lactic acid transport, but it also plays an important role in the invasion and metastasis of malignant tumor cells by stimulating the production of numerous matrix metalloproteinases (MMPs) and vascular endothelial growth factor by fibroblasts, and could also act as an autocrine factor stimulating MMPs production in metastatic tumor cells. Here, we found that silencing CD147 in chronically stressed nude mice not only inhibited the proliferation of xenografts but also decreased matrix metalloproteinase-2, 9 expression and lactic acid content in tumor tissues. Furthermore, norepinephrine (NE) was significantly increased in the serum of nude mice in glioma stress model. To determine the underlying cellular mechanism, we added exogenous NE into LN229 and U87 cells to simulate the stress environment *in vitro*. The invasiveness of the glioma cells was subsequently examined using a Matrigel invasion assay. We demonstrated that knockdown of CD147 inhibited glioma invasiveness and metastasis with norepinephrine stimulation. Luciferase reporter gene experiments further demonstrated that the expression of CD147 is up-regulated primarily by norepinephrine *via* the β-Adrenalin receptor (βAR)-β-arrestin1-ERK1/2-Sp1 pathway. High expression of CD147 promoted the secretion of MMP-2 and the increment of lactic acid, which accelerated the augmented invasion and metastasis of glioma induced by psychological stress. Taken together, these results suggest that psychological stress promotes glioma proliferation and invasiveness by up-regulating CD147 expression. Thus, CD147 might be a potential target site in the treatment of glioma progression induced by chronic psychological stress.

## Introduction

Glioma is the most common primary intracranial malignant tumor and is characterised by rapid growth, strong invasion potential and high recurrence rates (1). Psychological stress such as anxiety and fear are common in cancer patients. Intriguingly, clinical studies have found that psychological stress is closely related to the occurrence and prognosis of malignant tumors such as colon cancer (2), breast cancer (3), and chronic lymphocytic leukaemia (4). Long-term psychological stress can accelerate the proliferation and invasiveness of tumors such as lung cancer (5), breast cancer (6), and oral cancer (7) in tumor-bearing mice. Psychological stress leads to the continuous release of stress hormones, such as epinephrine (EPI) and norepinephrine (NE), which have a profound impact on both tumor progression and prognosis. Sood (8) investigated that stress levels of norepinephrine increased the *in vitro* aggressiveness of ovarian cancer cells and induced significant increases in invasion. Norepinephrine also increased tumor cell expression of MMPs. Consistent with this, the invasion ability of ovarian cancer cells was inhibited by the ß-adrenergic receptor antagonist propranolol hydrochloride. Yin (9) also found that norepinephrine increased the secretion of MMP-2 and promoted the migration and invasiveness of glioma T98G cells.

CD147 (also known as extracellular matrix metalloproteinase inducer, EMMPRIN) is widely expressed on the surface of a variety of tumor cells (10, 11). Importantly, CD147 can stimulate fibroblasts to produce diverse matrix metalloproteinases (MMPs) (12, 13). But it also can stimulate tumor cells to produce MMPs, specifically MMP-2 and MMP-9. MMPs can trigger the remodelling of basement membrane components and extracellular matrix molecules, which greatly facilitates tumor invasion, metastasis, and vascularisation (14). High expression of CD147 leads to increased tumor growth and metastasis in bone cancer cells and glioma, which is related to the induction of MMPs and modification of the tumor microenvironment (15, 16). Research has shown that high levels of stress hormones cause neuroimmunoendocrine system disorders (17), which leads to alterations in the tumor microenvironment (18). However, our previous study showed that activation of adrenergic receptors promoted the expression of LDHA, MCT1, and MCT4, all of which are directly related to the metabolism of lactic acid in glioma cells (19–21). The appropriate localization of MCT1 and MCT4 on the cell membrane requires the participation of CD147 (22). Baumann previously reported that lactic acid promoted the invasion and metastasis of malignant glioblastoma by inducing the expression of matrix metalloproteinase 2 (MMP-2) (23). This augmentation of the acidic microenvironment further promoted the proliferation and invasiveness in glioma cells. Many studies have shown that CD147 involves a variety of physiological or pathological processes, but it is not yet clear how CD147 participates in the regulation of psychological stress in gliomas. To investigate the role of CD147 on the proliferation and invasiveness of glioma induced by psychological stress, we used a nude mouse model of stress glioma in addition to cell proliferation and invasion assays and an *in vitro* fluorescein reporter gene experiment. Our study provides new ideas and targets for the psychological and drug intervention in the treatment of glioma.

## Materials and Methods

### Cell Culture

The human glioma cell line LN229 was obtained from Leibniz Institute DSMZ-German Collection of Microorganisms and Cell Cultures (Germany) and U87, U251, SHG44, and HEB cells were purchased from the Shanghai Institutes for Biological Sciences, Chinese Academy of Sciences (Shanghai China). They were cultured in Dulbecco’s Modified Eagle’sMedium (DMEM, GibcoBRL, Gaithersburg, MD, USA) supplemented with 10% foetal Bovine serum (FBS, Gibco BRL, Gaithersburg, MD, USA), 100 U/ml penicillin, and 100 µg/mL streptomycin at5% CO_2_ and 37°C.

### Construction of Short Hairpin RNA Targeting CD147

According to Chen’s research (24), and based on the CD147 cDNA sequence in GenBank, we used BLAST to design 3 pairs of 64 nucleotide oligos si-CD147-1, si-CD147-2, and si-CD147-3, with endonuclease restriction sites on both ends. We only wrote the most efficient sequence. The sequences used were: shRNA-CD147-1(GenBank:AB085790) 1-1 5’GATCCCCGTCGTCAGAACACATCAACTTCAAGAGAGTTGATGTGTTCTGACGACTTTTTGGAAA-3’, sequence1-2 5’-AGCTTTCCAAAAAGTCGTCAGAACACATCAACTCTCTTGAAGTTGATGTGTTCTGACGACGGG-3’; shRNA-control 2-1 5’CCACTACCGTTGTTATAGGTGTTCAAGAGACACCTATAACAACGGTAGTTTTTTTGGAAA-3’, sequence2-25’GCTTTTCCAAAAAAACTACCGTTGTTATAGGTGTCTCTTGAACACCTATAACAACGGTAGTG-3’. Two single strands of DNA complementary to shRNA CD147 were synthesized; the ends of the DNA were designed to contain the AgeI and EcoR I restriction sites and were annealed to form a double strand; This was ligated to pGV248-SC1 vector (purchased from Shanghai Jikai Biotechnology Co, LTD.). The lentiviral recombinant vector pGV248-SC1-CD147 was constructed, and HEK293T cells were used to package the virus. When the degree of fusion of glioma cells reached 80%, a cell suspension of 3x10^4^ cells/mL was prepared and seeded in 6-well plates. When the cell density reached 30%, the shRNA-control group and shRNA-CD147 group were transfected with empty lentivirus and shRNA-CD147 lentivirus, respectively. Cells were collected when the degree of cell fusion was approximately 80%, and interference effects were detected.

### Animal Models

All nude mice were pathogen-free and allowed access to food and water. Animal experiments were performed with the permission of the Animal Ethical Commission of Weifang Medical College. The skin on the lower right shoulder blade of nude mice was sterilized with alcohol and then a cell suspension of 5×10^7^ cells/ml in 0.2 ml DMEM was injected into each nude mouse. Mice were divided into the control group (Injection of DMEM medium) and the tumor stress group (Injection of LN229 cells), the shRNA-Con group (Injection of LN229 cells transfected with shRNA-control), the shRNA-CD47 group, and the shRNA-CD147 stress group (Injection of LN229 cells transfected with shRNA-CD147), with 5 mice in each group. The restraint stress mouse model (25) was established in the tumor stress group and the shRNA-CD147 stress group 5 days after injection of the cell suspension. The nude mice were placed in a 50 ml centrifuge tube with 3-mm-long holes. The animals were not squeezed during the restraint stress process. During the restraint stress period, mice were deprived of water and food. After 8 h of restraint stress, access to food and water was restored. The restraint stress process lasted 4 weeks and was performed 5 days per week. The sizes of subcutaneous tumors were measured daily. The tumor was removed and weighed after restraint stress. The long diameter (a) and short diameter (b) of the tumor were measured and the tumor volume was calculated (tumor volume =a x b^2^ x 0.5).

### Drug Administration

Mice with LN229 cells were randomly divided into four groups: Con + PBS (n = 5), Stress+PBS (n=5), Stress+ propranolol (n = 5) and Stress+ U0126 (n=5). Starting 24 h after tumor cell injection, mice were treated with daily i.p. injections of PBS (Con group), propranolol (2 mg/kg) (Stress+propranolol) (8) and U0126 (25 μmol/kg) (Stress+U0126) (26). All treatments were administered in a total volume of 200 AL. The restraint stress process lasted 4 weeks. The sizes of subcutaneous tumors were measured daily.

### Lactate Concentration Measurement

A portion of the tumor tissue was removed and washed twice with ice cold 1x PBS after the mice were sacrificed. The tumor tissues were homogenized in ice cold 1x PBS with a proportion of mass (g): volume (ml)=1:9, and then centrifugated at 2,500 rpm for 10 min after which the supernatant was collected. LN229 cells were washed twice with PBS, cultured in DMEM without glucose, pyruvate and serum for 30 min (Le et al., 2011), and then incubated with Propranolol (10 μmol/L) or U0126 (10 μmol/L) for 30 min. Cell culture medium (1 ml) was collected with NE(10 μmol/L) treatment after 24 h, then centrifuged at 2,500 rpm for 10 min after which the supernatant was collected. The same volume of supernatant was used for each group for the lactic acid determination kit (Abcam; Ab65331). Moreover, the protein content was determined by the BCA method to calculate the quantity of lactic acid.

### EPI and NE Concentration Measurement

After restraint stress was completed, the eye of the mouse was removed and the blood was allowed to flow from the eye socket into a tube, which was placed at 4°C for 12 h. The supernatant was then centrifuged at 1000 rpm for 20 min. The EPI and NE concentrations were measured using an EPI, NE ELISA kit, respectively (Rapid Bio Lab).

### Transfection of Cells

LN229 and U87 cells were plated in six-well plates at a density of 2×10^5^ cells per well and incubated overnight. The cells were transfected with different interference; the interference sequence (27, 28) is 5’-GAAGUCAAAGCCUUCUGCGCGGAGA-3, siRNA-β-arrestin2, 5’-CCUGAAGGACCGCAAAGUGUUUGUG-3’, Sp1,5’-AAUGAGAACAGCAACAACUtt-3’, and siRNA-Con using Lipofectamine 2000 (Invitrogen, Carlsbad, CA, USA) according to the manufacturer’s instructions.

### Quantitative Real Time Polymerase Chain Reaction (qPCR)

qPCR was performed using an ABI Prism 7500 fluorescence quantitative PCR instrument (Applied Biosystems, Foster City, CA, USA). An amplification mixture was prepared using a SYBR^®^ Premix Ex Taq™ Kit (Takara, Dalian, China) according to the manufacturer’s protocol. The cycling parameters were 2 min at 95 °C, followed by 30 cycles of 10 s at 95 °C, 30 s at 60 °C and 30s at 72 °C. Reactions were prepared in duplicate for each sample. The expression levels of endogenous markers were quantified and are presented as the mean cycle threshold (Ct) values, as detected with sequence-detection system software v2.3 (Applied Biosystems) using a threshold value of 0.2. The primers were as follows: CD147 forward, 5’-GCAGCGGTTGGAGGTTGT-3’, and reverse, 5’-AGCCACGATGCCCAGGAAGG-3’; MMP-2 forward, 5’-AGAGACAGTGGATGATGCCTTT-3’, and reverse, 5’-ATCGTCATCAAAATGGGAGTCT-3; MMP-9 forward, 5’-TGGGGGGCAACTCGG-3’, and reverse,5’-GGAATGATCTAAGCCAG-3; β-actin forward, 5’-AGGGGCCGGACTCATCGTA-3’, and reverse, 5’-GAGCTCACCATTCACCATCTTGTC-3.

### Western Blotting

The same amount of protein was loaded onto an SDS-PAGE gel, transferred to a nitrocellulose (NC) membrane, and blocked with 50 g/L non-fat milk for 1 h. The membrane was then incubated with the following antibodies at 4°C: rabbit anti-CD147 antibody (MAB972-100, R@D, 1:3,000) and rabbit anti-ERK1/2 antibody (Cell Signaling Technology, 1:5,000), rabbit anti-phospho-ERK1/2 antibody (Cell Signaling Technology, 1:5,000), rabbit anti-Sp1 antibody (sc-14027, Santa Cruz Biotechnology, 1:2,000), rabbit anti-MMP2 antibody (ab86607, abcam, 1:3,000) rabbit anti-MMP9 antibody (ab236494, abcam, 1:2,000), and mouse anti-β-Actin (sc-8432; Santa Cruz Biotechnology,1:5,000). The NC membrane was washed with TBST 3 times for 10 min each time and was then incubated with a secondary antibody at room temperature for 1.5 h, followed by 3 washes in TBST. The membrane was then treated with enhanced chemiluminescence (ECL) reagent. The membrane was scanned and the absorbance (A) values of the protein bands were analysed.

### Proliferation Assays

Cells in the logarithm growth phase were inoculated into 96-well plates at a density of 3x10^3^ cells per well. Cells were divided into five groups: Con (LN229 or U87 cells), NE (LN229 or U87 cells with 10 μmol/L NE), shRNA Con (transfected with shRNA-Con), shRNA-CD147 (transfected with shRNA-CD147), and shRNA-CD147 +NE (transfected with shRNA-CD147 LN229 and U87 cells treated with 10 μmol/L NE). The absorbance value of the cells was obtained by reading the absorbance (A) values of 450 through a multi-well spectrophotometer after cells were incubated for 4 h with CCK-8 reagent (Dojindo, Kumamoto, Japan) at different time points. A standard curve was obtained by plotting cell numbers against absorbance values; DMEM was used as a control.

### Matrigel Invasion Assay

100 μl (1:10 Sigma, USA) and 300 μl precooled serum-free DMEM (Gibco) were mixed. 24 h after transfection, LN229 or U87 cells were resuspended in DMEM (serum-free) and added (1.25 × 10 ^5^/100 µl) to each Matrigel-coated upper chamber, which had been incubated with 50 μl of prepared matrix glue (Corning NY, USA), at room temperature for 1 h. Moreover, 10 μmol/L NE was added to the cell suspensions of the NE and shRNA-CD147 +NE groups, respectively. Then, 600 μl fresh DMEM containing 10% FBS was added to the lower chamber. After 24 h, cells that had not migrated were removed and the inserts were fixed in 4% paraformaldehyde for 15 min, stained with 0.2% crystal violet for 20 min and rinsed 3 times in PBS. Five different fields were observed, the mean value (200X) was calculated, and 3 independent experiments were performed. Data are presented as the mean ± SD.

### Migration Assays

Glioma cells were seeded onto a 6-well plate. Upon reaching confluency, a scratch was made using a sterile pipette tip. To remove non-adherent cells, the wells were washed twice with medium and subsequently imaged. Cell migration was observed and photographed at 0 and 24 h Cell migration at the wound edge was quantified and presented as the mean ± SD.

### Gelatine Zymography Assay

The supernatant was harvested and mixed with sample buffer (in the absence of reducing agents) and electrophoresed in a 1 mg/ml gelatine (SIGMA-G1890, USA) containing SDS-PAGE gel. Next, the gel was washed twice in wash buffer (2.5% Triton X-100, 50 mmol/L Tris-HCl, 5 mmol/L CaCl2, pH 7.6), rinsed two times with 50 mmol/L Tris-HCl, 5 mmol/L CaCl2, pH 7.6, and incubated in 50 mmol/L Tris-HCl, 5 mmol/CaCl_2_, 0.02% Brij-35(P5884, Sigma-Aldrich, USA), pH 7.6 for 42 h. The gel was then stained with 0.25% Coomassie blue. Proteolysis was detected as a white zone in a dark blue field.

### CD147 Reporter Constructs and Luciferase Assay

According to the Literature (29), the CD147 promoter was amplified from mouse genomic DNA using primers and PCR to generate a 1131-bp product containing KpnI (5-end) and HindIII (3-end) restriction sites. CD147 was cloned into pGL4.16 (Promega, Madison WI, USA) to generate pGL4.16-CD147- P. Other reporter plasmids were produced by truncations using the pGL4.16-CD147- P construct as a template. The primers used for sub-cloning are listed in **Table 1**. For the luciferase reporter assay, cells were co-transfected with the specific pGL4.16-CD147 luciferase plasmid (wild type, different truncations or mutants), transcriptional factor 1 (Sp1) expression plasmids and the pRL-TK renilla plasmid using Lipofectamine 2000. After transfection for 30 h, the cells underwent suitable treatments with the adrenaline agonist NE (10 μmol/L) for 8 h, after which the luciferase activities were measured using a dual-luciferase reporter assay system (E1910, Promega, Madison WI, USA) according to the manufacturer’s instructions. The luciferase activity levels were calculated as the ratio of firefly luciferase activity to renilla luciferase activity, and the results are presented as the average of at least triplicate experiments.

**Table 1:** Oligonucleotide sequence of polymerase chain reaction (PCR) primers.

Oligo names	Forward	Reverse
pGL4.16-CD147-M4(-1080/+51)	5’-ATCGGGTACCCAAGAAAGGTAACCGCCAGC-3’	5’-ATCGAAGCTTCAGCGCAGCCGCCATGATTC-3’
pGL4.16-CD147-M3(-1080/+51)	5’-ATCGGGTACCTCCAGCCGCGTCCCCGCAG-3’	
pGL4.16-CD147-M2(-1080/+51)	5’-ATCGGGTACCCTGCCGGAGCCGGCGCGTAC-3’	
pGL4.16-CD147-M1(-1080/+51)	5’-ATCGGGTACCACCGGCGTCCCCGGCGCTC-3’	
pGL4.16-CD147(-1080/-240)	5’-ATCGGGTACCCCGCTTTTTATAGCGGCCGCGG-3’	
pGL4.16-CD147(-35/+51)	5’-ATCGAAGCTTCTGGAGCCTGCGCATTTCTTCC-3’	5’-ATCGGGTACCCAAGAAAGGTAACCGCCAGC-3’
pGL4.16-CD147(-106/+51)	5’-CTGCCGCCGCCGTTTTCGGCCTATAA-3’	5’-TTATAGCGGCCGAAAACGGCGGCGGCAG-3’
pGL4.16-CD147(-160/+51)	5’-CATCTCGGGGGCGGTTTTAGCGCCGGGGAC-3’	5’-GTCCCCGGCGCTAAAACCGCCCCCGAGATG-3’
pGL4.16-CD147(-245/+51)	5’-GCCGGTCGCCCCGTTTTCGCACGCGCGCAC-3’	5’-GTGCGCGCGTGCGAAAACGGGGCGACCGGC-3’
pGL4.16-CD147-P1(-1080/+51)	5’-CTGCCGCCGCCGTTTTCGGCCGCTATAA-3’	5’-TTATAGCGGCCGAAAACGGCGGCGGCAG-3’

### Site-Directed Mutagenesis

CD147 promoter mutations (CD147-M1, CD147-M2, CD147-M3, and CD147-M4) were generated using the QuickChange mutagenesis kit obtained from Stratagene. All mutations were verified by DNA sequencing.

### Statistical Analysis

SPSS16.0 statistical software was used for the analysis. All data are presented as the means ± SD of at least three independent experiments. Statistical comparisons were performed using ANOVA, and a t-test was used for pairwise comparisons between groups. P<0.05 was considered statistically significant.

## Results

### Silencing CD147 Inhibited the Growth of Transplanted Tumors in Nude Mice Augmented by Psychological Stress

**Figure 1:**
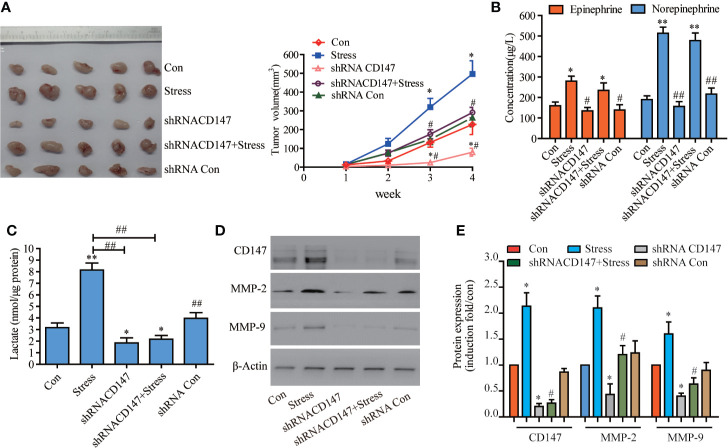
CD147 Silencing Inhibited the Proliferation of Xenograft Tumors Induced by Chronic Psychological Stress in Nude Mice. **(A)** The growth of transplanted tumors in nude mice (n=5). **(B)** epinephrine (EPI) and norepinephrine (NE) concentrations in the serum of nude mice with transplanted tumors were detected by ELISA, and the stress hormone levels were not affected after CD147 silencing. **(C)** Determination of lactic acid in the tumor tissues of nude mice. The expression of CD147, MMP-2, and MMP-9 in tumor tissues of nude mice were detected by western blotting. **(D)** Evaluated by Western blots and **(E)** Quantified. (left to right). CD147, MMP-2, and MMP-9 expression levels were increased in nude mice with glioma and psychological stress treatment. Data are expressed as the mean ± sd. *P < 0.05. **P < 0.01 vs Con. ^#^P < 0.05. ^##^P < 0.01 vs Stress.

Immunohistochemistry analysis revealed that the vast majority of 78 paraffin-embedded archival glioma specimens tested displayed positive CD47 expression, CD147 had stronger staining in high-grade glioma tissues than low grade glioma tissues (**Supplementary Figure 1A**). Patients with low CD147 expression had higher overall survival in high-grade gliomas than those with high CD147 expression (**Supplementary Figure 1B**). We analysed CD147, its transcriptome in data from 603 glioma patients using the Chinese Glioma Genome Atlas (CGGA) was collected and identified, database (http://www.cgga.org.cn/download_other.jsp). High-grade glioma patients with high levels of CD147 exhibited poorer overall survival rate compared with those with low CD147 levels (**Supplementary Figures C, D**). Psychological stress has been shown to promote proliferation and invasiveness in a variety of tumor cells (30). In stressed nude mice with glioma, we found that the tumor proliferation rate was increased significantly, while the tumor growth rate of psychological stress-induced transplantation was reduced by 50.63% after CD147 knockdown (**Figure 1A**, F=1.565, P=0.0014, n=5). Serum EPI and NE concentrations in the stressed group were significantly higher than those in control group. Remarkably, serum NE was elevated 2.8 times in nude mice with psychological stress (**Figure 1B**). We also demonstrated that the lactic acid concentration in tumor tissues was increased significantly in the stressed group but was decreased by 82.16% after CD147 silencing (**Figure 1C**, F=25.00, P=0.0166, n=5). CD147 not only plays a role in lactic acid transportation, but it also can stimulate tumor cells to produce MMPs, specifically MMP-2 and MMP-9, which further promotes tumor invasion and metastasis (31). We found that the effects of CD147 on glioma cancer cells were mediated by stimulating the synthesis of MMP-2 and MMP-9. The expression of CD147, MMP-2, and MMP-9 proteins were also increased significantly in the stressed group and decreased in the CD147 silenced group (**Figures 1D, E**).

### NE Promoted the Expression of CD147 and MMP-2 in Glioma Cells

**Figure 2:**
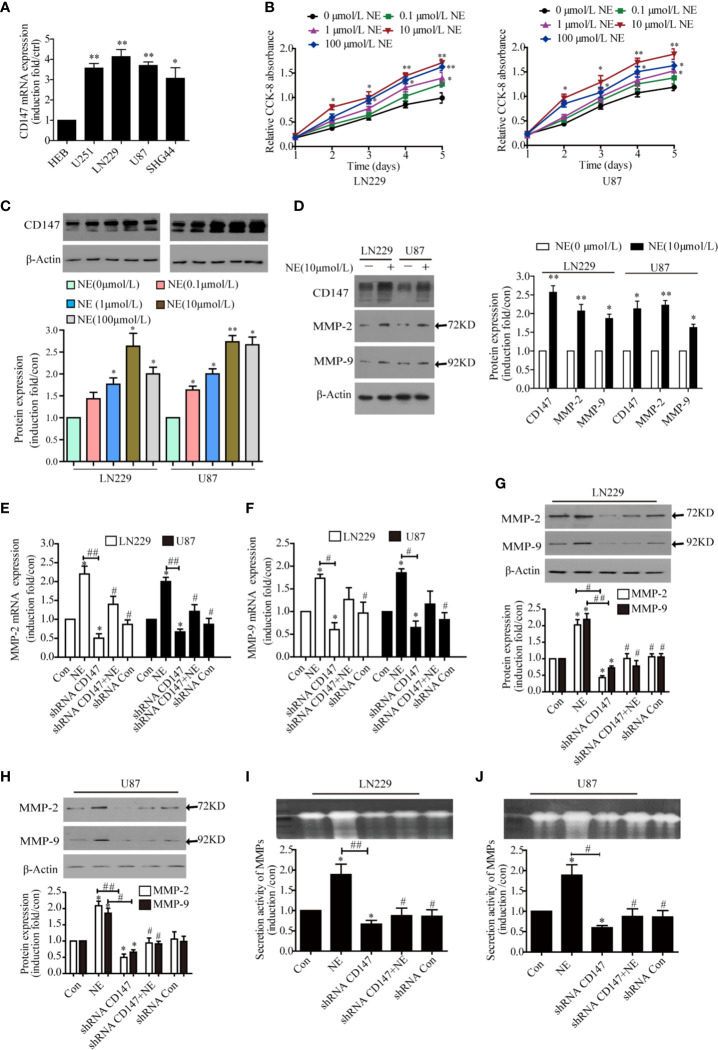
CD147 Knockdown Reduced the Expression and Secretion of MMP-2 and MMP-9 Induced by norepinephrine (NE) in glioma Cells. **(A)** Detect the relative expression of CD147 in human glioma cell lines including HEB, U251, LN229, U87, and SHG44 by PCR. **(B)** Two glioma cells were incubated with different concentrations of NE for 1, 2, 3, 4, and 5 day. CCK-8 assay demonstrated that NE promoted the proliferation of glioma cells in a concentration-dependent and time-dependent manner. **(C)** NE increased expression of CD147 in a concentration-dependent manner. **(D)** The expression of CD147, MMP-2, and MMP-9 proteins was detected by western blotting. NE promoted the expression of CD147, MMP-2, and MMP-9. **(E, F)** qPCR confirmed that NE promoted the expression of MMP-2 and MMP-9, which were inhibited by CD147 knockdown. **(G, H)** Western blotting analysis showed that the expression of MMP-2 and MMP-9 protein in LN229 cells and U87 cells were decreased after transfection with shRNA-CD147. **(I, J)** Gelatinase assay showed that NE promoted matrix metalloproteinases (MMPs) secretion in two glioma cells, which was inhibited by CD147 knockdown. shRNA-Con was the negative control; data are expressed as the mean ± SD. *P < 0.05. **P < 0.01 vs Con (0 μmol/L NE). ^#^P < 0.05. ^##^P < 0.01 vs 10 μmol/L NE.

NE was used to simulate adverse psychological stress in glioma cells (8). qPCR experiments showed that CD147 expression was increased in glioma cell lines comparing with normal cell line HEB. The highest expression level of CD147 were found in LN229 and U87 cells, so they were selected for further experiments (**Figure 2A**). Various concentrations of NE (0. 0.1, 1, 10, 100 μmol/L) were applied to glioma cells for 1, 2, 3, 4, and 5 day. All tested concentrations of NE promoted glioma cell proliferation compared with the control (0 μmol/L NE), especially 10 μmol/L NE. Cell proliferation is concentration and time dependent manner (**Figure 2B**). Cells were treated with differere concentrations of NE for 16 h, These results demonstrate that NE-induced expression of CD147 is concentration-dependent manner (**Figure 2C**). Our work found that NE promoted the expression of CD147, MMP-2, and MMP9 (**Figure 2D**). CD147 can stimulate tumor cells to produce MMPs, specifically MMP-2 and MMP-9, especially in metastatic tumor cells (32). Kaji T (33) showed that MMP-2 and MMP-9 were highly expressed in glioma cells. Zhang (34) also reported similar results. To further investigate the effect of psychological stress on the invasion potential of glioma cells, we examined the expression and secretion of MMP-2 and MMP-9. Our results revealed that MMP-2 and MMP-9 expression in glioma cells were increased at both the RNA and protein levels after NE treatment, whereas CD147 silencing inhibited MMP-2 and MMP-9 expression (P<0.05) (**Figures 2E–H**). Gelatinase analysis showed that NE treatment of LN229 and U87 cells increased the secretion of MMPs, whereas CD147 silencing inhibited this effect (**Figures 2I, J**). These data suggest that NE promotes the invasion of glioma cells by inducing the expression and secretion of MMP-2, MMP-9 and that silencing CD147 can reverse the expression and secretion of MMP-2 and MM-9 protein induced by NE.

### CD147 Knockdown Inhibited Clone Formation, Migration, and Invasiveness of Glioma Cells Induced by NE

**Figure 3:**
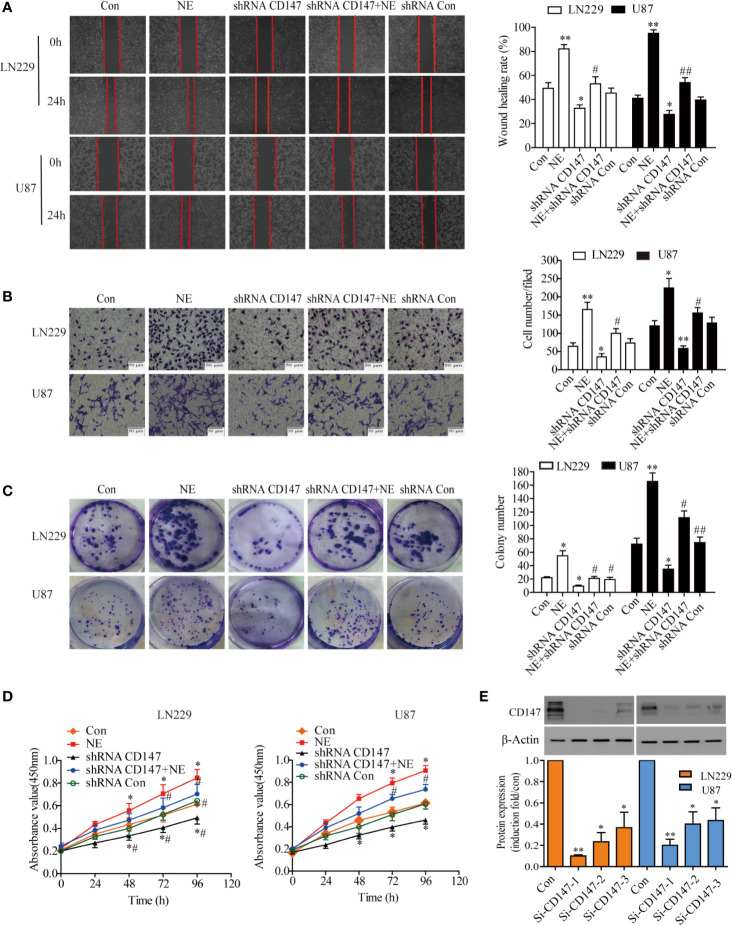
CD147 Knockout Inhibited the Proliferation, Invasiveness, and Migration of LN229 and U87 Cells. **(A)** Migration ability of LN229 and U87 cells were detected by scratch assay. **(B)** Representative images of the invasion ability of and U87 cells [control group, norepinephrine (NE) group, shRNACD147 group, shRNA CD147 + NE group and shRNA Con] in the Matrigel invasion assays (*×*200). Quantification of invaded cells showed that interference with CD147 expression significantly inhibited the invasion of Matrigel in NE -induced LN229 and U87 cells. **(C)** Colony formation assay. Interference with CD147 expression significantly inhibited the formation of LN229 and U87 cell clones induced by NE (10 μmol/L). **(D)** CCK-8 assay (at 0, 24, 48, 72, and 96 h) in two glioma cells induced by NE(10μmol/L). CD147-interference significantly inhibited the proliferation of two glioma cells induced by NE. **(E)** Detection of CD147 silencing effect by Western blot and quantified. Data are expressed as the mean ± sd. *P < 0.05. **P < 0.01 vs Con (0 μmol/L NE); ^#^P < 0.05. ^##^P < 0.01 vs 10 μmol/L NE.

CD147 is widely expressed on various tumor cell surfaces and plays a role in tumourigenesis and invasion. Therefore, we next investigated the effect of silencing CD147 on clone formation, migration and invasiveness of LN229 and U87 cells induced by NE (10μmol/L). Cell migration was first determined by scratch test. The results showed that the migration rate of LN229 cells was increased significantly after NE (10 μmol/L) treatment for 24 h and that this effect was decreased after CD147 was silenced (**Figure 3A**). Our results demonstrated that NE promoted the invasiveness of glioma cells, while the number of cells that migrated through the matrix glue in the shRNA-CD147 group of LN229 cells decreased by 43.75% and that the shRNA-CD147 group of U87 cells were reduced by 53.57% compared with the control group (P<0.05) (**Figure 3B**). In order to further study the effect of silencing CD147 on the proliferation of glioma cells, clonogenic and CCK-8 cell proliferation experiments were conducted. These results showed that the proliferation ability of two glioma cells were increased after NE (10 μmol/L) treatment and that their colony formation ability was also significantly increased (P<0.05). In the shRNA-CD14**7** group**, c**olony formation ability was also significantly reduced (**Figure 3C**) and proliferation ability was decreased (**Figure 3D**). CD147 expression decreased with si-CD147-1, si-CD147-2, and si-CD147-3 in two glioma cells. Cells were transfected with si-CD147-1, which had the most effective interference effect among them (**Figure 3E**).

### The Stress Hormone NE Regulated CD147 Protein Expression Through the βAR-Beta Arrestin1-ERK Pathway

**Figure 4:**
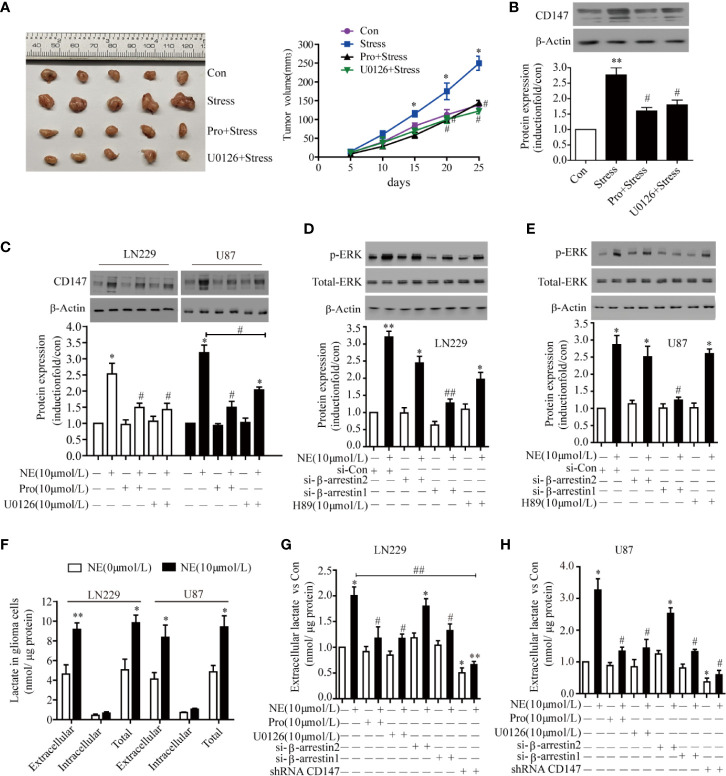
The β-adrenergic Receptors/β-arrestin1/ERK Signaling Pathway Regulated CD147 Expression. **(A)** Propranolol and U0126 reduced tumor size *in vivo*. **(B)** U0126 and Propranolol blocked the expression of CD147 induced by stress. **(C)** U0126 and Propranolol blocked norepinephrine (NE)-induced CD147 expression. **(D)** ERK1/2 signaling regulated CD147 expression in LN229 and **(E)** U87 cells downstream of β-AR. Cells were treated with NE (10 μmol/L) for 16 h. The effect of U0126 (10 μmol/L) on NE-induced CD147 expression was monitored by western blotting and quantified. **(F)** The production of lactic acid in extracellular and intracellular compartments of glioma cells under the effect of NE. The ERK1/2 inhibitor U0126 (10 μmol/L) inhibited NE-induced lactate release in LN229 cells **(G)** and U87 cells **(H)**, which was also inhibited by propranolol, shRNACD147, and si-β-arrestin1. Data are expressed as the mean ± SD. *P < 0.05. **P < 0.01 vs NE (0 μmol/L); ^#^P < 0.05. ^##^P < 0.01 vs NE (10 μmol/L) or stress.

Next, we investigated the receptor that mediated CD147 up-regulation by NE. Propranolol and U0126 reduced tumor size *in vivo*. Tumor volume was significantly increased in stress group. Treatment with propranolol blocked the stesss-induced increase in tumor volume reduce. The tumor size was significantly reduced in propranolol +stress or U0126+ stress group. Compared with the stress group, the volume of U0126+ stress group and Pro+stress group tumors decreased (P<0.05) (**Figure 4A**). The expression of CD147 was increased in the stress group, while U0126 and Pro inhibited the expression of CD147 (**Figure 4B**). Two glioma cells were incubated with propranolol (10 μmol/L), an inhibitor of the β-adrenergic receptor (βAR), and U0126 (10 μmol/L), an ERK inhibitor, for 30 min; the cells were then incubated with 10 μmol/L NE for 16 h. Both propranolol and U0126 were sufficient to block the promoting effect of NE on the expression of CD147 (**Figure 4C**). β-adrenergic receptors can bind to different downstream molecules, such as PKA and β-arrestin, and activate diverse signaling pathways to regulate cell function. The level of endogenous β-arrestin1 was down-regulated, which reduced the level of ERK phosphorylation induced by 10 μmol/L NE (P<0.05), but the level of ERK phosphorylation was not inhibited by either beta-arrestin 2 silencing or the PKA inhibitor H89 (P>0.05) (**Figures 4D, E**). These results suggest that NE regulates CD147 protein expression through the βAR/beta-arrestin1/ERK pathway. CD147, as a chaperone of lactate transporters, is involved in lactic acid transport. Lactic acid (a tumor microenvironmental stress factor) is the main substrate for the formation of an acidic microenvironment surrounding a tumor, which promotes the proliferation, metastasis and invasiveness of tumor cells (35). Futhermore, incubation with 10 μmol/L of NE promoted increased the total lactate level in 24 h in LN229 and U87 cells(P < 0.05), It had no effect on intracellular lactic acid in the cells in 24 h(P > 0.05). Most of the lactate produced by the NE-induced lactic acid was transported outside the cells (**Figure 4F**). Glioma cells were incubated with NE (10 μmol/L) for 24 h after interfering with beta-arrestin1 expression for 30 h. The results show that the amount of extracellular lactic acid was significantly reduced and that both propranolol and U0126 could significantly reduce the amount of lactic acid in two glioma (**Figures 4G, H**).

### The Transcription Factor Sp1 Mediated NE-Induced Transcription of CD147

**Figure 5:**
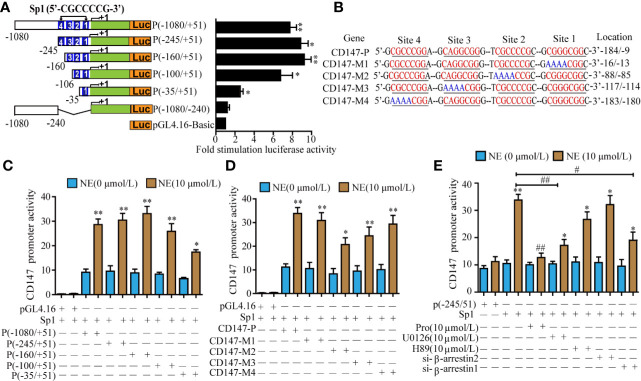
The Transcription Factor Sp1 Mediated the norepinephrine (NE)-Induced High Expression of CD147. **(A)** Schematic diagram of luciferase assay for different lengths of CD147 promoter activity, *P<0.05, **p<0.01 compared with the empty PGL4.16 vector. **(B)** Luciferase activity of different truncated human CD147 promoters after stimulation with 10 μmol/L NE. **(C)** Schematic diagram of the mutation in the node of Sp1 in the CD147 promoter. **(D)** Detection of 10 μmol/L NE-induced luciferase activity of CD147-P, CD147-M1, CD147-M2, CD147-M3, and CD147-M4 co-transfected with Sp1. **(E)** The effect of the β-adrenergic receptor inhibitor propranolol (10 μmol/L), the PKA inhibitor H89 (10 μmol/L), the ERK inhibitor U0126 (10 μmol/L), si-β-arrestin1, and si-β-arrestin2 on Sp1- mediated CD147-P (-245/+51) luciferase activity. Luciferase activity was normalized to that of renilla luciferase, *P < 0.05, **P < 0.01, compared with 0μmol/L NE; ^#^P < 0.05, ^##^P < 0.01 compared with Sp1-CD147-P (-245/+51)+10 μmol/L NE.

To study the minimum promoter region where NE regulates CD147 transcription in LN229 cells, we constructed a series of 5 ‘-deficient promoters that were fused into firefly luciferase and evaluated their activity in LN229 cells. When the -1080 to -245 regions were deleted, NE triggered increased luciferase activity, which suggests that this is the negative control element in the promoter region. When the -245 and -106 (site 4) regions were deleted, CD147 promoter activity showed no significant change. The knockout of sequences between -245 and -106 (sites 4, 3) resulted in reduced CD147 promoter activity. The promoter activity of CD147 was significantly reduced after the deletion of sequences between -245 and -9 (sites 1, 2, 3, 4). Therefore, NE induced CD147 transcription by binding primarily to the sequence between -160 and -9 (**Figures 5A, C**). The CD147 promoter region contains four Sp1 binding regions between -245 and -9, and Sp1 is considered to be critical for NE regulation of CD147 promoter activity. After co-transfection of LN229 cells with Sp1 and CD147-P luciferase reporter genes of different lengths, we found that the 1, 2, and 3 mutations significantly reduced CD147 promoter activity. These results demonstrate that the transcription of CD147-P1 induced by NE occurs when Sp1 binds primarily to sites 1, 2, and 3 (**Figures 5B, D**). Sp1-mediated CD147-P luciferase activity was significantly reduced by either propranolol, U0126, or si-β-arrestin1 (P<0.05), while the PKA inhibitor H89 and si-β-arrestin2 had no significant effect on CD147-P promoter activity (P>0.05) (**Figure 5E**). These findings suggest that NE regulates CD147 expression by activating the β-Adrenergic receptor/β-arrestin1/ERK1/2 -Sp1 pathway.

### NE Regulated CD147 Expression by Activating the β-AR/β-Arrestin1/ERK/Sp1 Pathway

**Figure 6:**
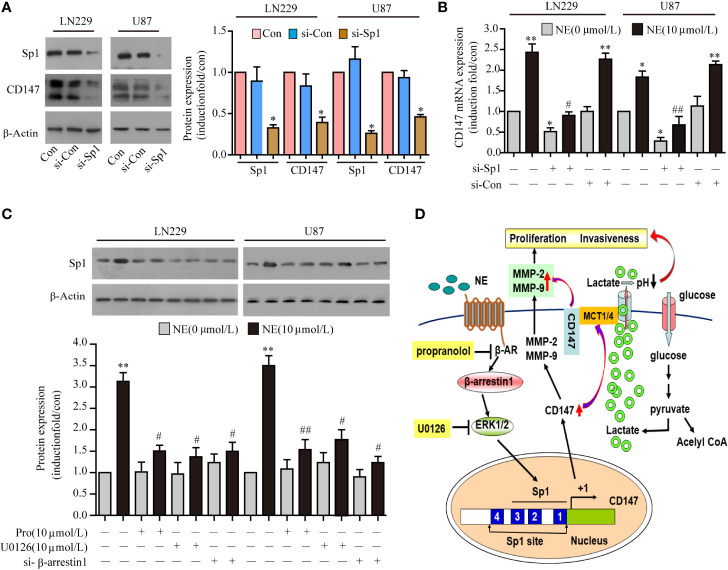
Norepinephrine (NE) Regulated CD147 Expression by Activating the β-AR/β-Arrestin1/ERK/Sp1 Pathway. **(A)** Expression of CD147 and Sp1 protein and statistical analysis **(B)** and mRNA in LN229 and U87 cells after transfection with Sp1 siRNA. **(C)** Effects of U0126, si-β-arrestin1 or Propranolol on NE-induced Sp1 expression in LN229 and U87 cells. **(D)** Schematic diagram of how NE regulates the expression of CD147 through the β- adrenergic receptor-β-arrestin1-ERK-Sp1 pathway. *P < 0.05, **P < 0.01, compared with 0 μmol/L NE; ^#^P < 0.05, ^##^P < 0.01 compared with 10 μmol/L NE.

The underlying mechanism of how NE activates CD147 is not completely clear. The upstream sequence of the CD147 promoter contains an Sp1 protein binding site, which is also the binding site for adrenergic receptors. Therefore, we speculated that NE was likely to act on the binding site of Sp1 within the CD147 promoter through the adrenergic receptor mediated signaling pathway that would induce the expression of CD147. We found that the expression of CD147 induced by NE (10 μmol/L) was significantly reduced through Sp1 knockdown in two glioma (**Figures 6A, B**). While Sp1 expression was blocked by propranolol (10 μmol/L), the ERK inhibitor U0126 (10 μmol/L), and si-β-arrestin1 (**Figure 6C**). This suggested that NE may regulate CD147 expression by activating the β-adrenergic receptor-beta-arrestin1-ERK-Sp1 pathway. Taken together, these data indicate that the β-adrenergic receptor-β-arrestin1-ERK-Sp1 signaling pathway is required for the increase in CD147 expression induced by NE see the model (**Figure 6D**).

## Discussion

Glioma is one of the most common malignant tumors of the central nervous system. Its characteristic invasive growth leads to treatment difficulty and a high recurrence rate. Since medical models have shifted from biomedical models to biopsychosocial models, the impact of psychosocial factors on cancer patients has received increased attention.

Patients with cancer are always under adverse psychological stress. This leads to activation of the sympathetic-adrenal medullary axis and a subsequent increase in the plasma concentrations of adrenaline and norepinephrine. Studies have shown that norepinephrine can promote the proliferation and invasiveness of different tumor cells *via* distinct mechanisms. Liu found that NE could promote the occurrence and development of colon cancer by up-regulating the expression of vascular endothelial growth factor (35). In the mouse fibroblast 3T3 cell line, NE was reported to be associated with tumorigenicity by inducing DNA damage (36). It has been reported that NE can trigger epithelial mesenchymal transition in lung cancer cells and promote the invasion and metastasis of lung cancer by regulating the transforming growth factor-beta 1 signaling pathway (37). Our previous studies have found that NE promotes the production of lactic acid through the beta-adrenergic receptor, which leads to acidification of the extracellular space, thus creating a favourable environment for the proliferation and metastasis of glioma cells (19). The generation and transport of lactic acid depend on the participation and assistance of CD147 (10, 38). Our study demonstrated that NE promoted the expression of CD147, which resulted in an increase of lactic acid in the supernatant of glioma cells, whereas silencing CD147 reduced the proliferation and metastasis of glioma cells. Our findings reveal one of the mechanisms underlying the process whereby psychological stress augments the invasiveness of glioma cells.

MMPs play an important role in the invasion and metastasis of malignant tumors by promoting the degradation of the extracellular matrix and promoting tumor angiogenesis (39, 40). CD147 can stimulate the production of MMPs in mesenchymal cells surrounding the tumor, Bencivenga found that CD147 could stimulate MMP secretion and induce the expression of vascular endothelial growth factor in glioma cells, thus promoting tumor invasion and metastasis (41). Our data demonstrate that CD147 acts both as an autocrine factor stimulating MMP production and secretion in glioma cells, and as a paracrine factor regulating MMP production by endothelial cells, thereby facilitating tumor cell dissemination to secondary sites. That NE up-regulated the expression of CD147. This increased expression of CD147 then induced the secretion and expression of MMP-2 and MMP-9. Importantly, interference with CD147 expression significantly reduced the secretion and expression of MMP-2 and MMP-9, which reduced the proliferation of glioma cells induced by psychological stress. Although the role of CD147-induced MMPs expression in tumor invasion and metastasis has been reported, the underlying mechanism of the regulation of CD147 expression has not been well described.

EPI and NE perform their functions mainly through the adrenergic receptors on the surface of target cells. Adrenaline receptors were first divided into alpha and beta subtypes by Alquist in 1948. The endogenous ligands adrenaline and norepinephrine activate a series of signaling cascades of key proteins such as PKA. In addition to PKA, phosphorylated beta ARs recruit beta-arrestins, which then activate different downstream molecules such as Src and ERK, and thus they regulate a variety of cellular functions (42). NE affects the invasion and migration ability of various tumor cells, such as ovarian cancer and gastric cancer cells, mainly through beta receptors (43, 44). In recent years, important progress on the regulation of beta receptors in different pathological processes has been reported. For example, a study on beta-arrestin1 knockout mice revealed that under chronic stress, DNA damage and genomic instability resulted from the β-AR-beta-arrestin1 pathway (12). Our study found that CD147 was up-regulated by β-AR under NE stimulation, but no obvious increase in MCT1 and MCT4 was observed, this result is not shown in the article. However, Formoterol increases the expression of MCT1 and MCT4 through GS and β-arrestin1 aignal (19, 21), and different stress hormones may regulate the expression of lactate transport-related proteins through different mechanisms and jointly regulate the tumor microenvironment. Here, we confirmed CD147 as a direct target of Sp1 in glioma using bioinformatics binding site prediction, a dual luciferase reporting system and western blot analysis. Inhibition of Sp1 significantly reduced the NE-induced CD147 transcription level, which suggests that Sp1 plays an important role in the stress response and that this process can be blocked by propranolol, U0126, and si-β-arrestin1.

Taken together, these data suggest that psychological stress regulates CD147 expression though the β-AR-arrestin1-ERK-Sp1 pathway, which is activated by β- AR in the LN229 and U87cells line; this promotes the transport of lactic acid and the expression and secretion of MMP-2, MMP-9, and thereby augments the proliferation and invasion capabilities of glioma.

## Conclusion

The expression of CD147 is up-regulated primarily by Stress hormone NE *via* the β-Adrenalin receptor (βAR)-β-arrestin1-ERK1/2-Sp1 pathway. High expression of CD147 promoted the secretion of MMP-2 and the level of extracellular lactic acid, which accelerated the augmented invasion and metastasis of glioma induced by psychological stress. Knockdown of CD147 inhibited cell proliferation, migration, and invasion *in vitro*. Thus, CD147 might be a potential target site in the treatment of glioma progression induced by chronic psychological stress.

## Data Availability Statement

The original contributions presented in the study are included in the article/**Supplementary Material**; further inquiries can be directed to the corresponding author.

## Ethics Statement

The animal study was reviewed and approved by Medical Ethics Committee of Weifang Medical College.

## Author Contributions

PW and ZW contributed to the study design and the acquisition, analysis and interpretation of data. LX, WT, MQ, YY, AM, FS, and GL contributed key experimental materials and contributed to data analysis and manuscript revision. JD contributed to study conception and design, the analysis and interpretation of data, and drafting/revising the article. All authors contributed to the article and approved the submitted version.

## Funding

This work was supported by a project grant from The National Natural Science Foundation of China (81500798 to JD and 81871892 to MQ), the Natural Science Foundation of Shandong Province (ZR2014JL050 to JD), the Weifang Medical College Doctoral Start Fund (2017BSQD45 to JD), the Weifang Municipal Health and Family Planning Commission Scientific Research Project (2017wsjs103 to ZW), and the National innovation training program for college students (201910438002 to YY).

## Conflict of Interest

The authors declare that the research was conducted in the absence of any commercial or financial relationships that could be construed as a potential conflict of interest.
