# Supplementary material for: Psychological Stress Up-Regulates CD147 Expression Through Beta-Arrestin1/ERK to Promote Proliferation and Invasiveness of Glioma Cells
Source: Front Oncol. 2020 Oct 15;10:571181. doi: 10.3389/fonc.2020.571181 (PMC7593686; doi:10.3389/fonc.2020.571181)
Supplement: Supplementary file 1 [file DataSheet_1.pdf]

Supplementary Material

Supplementary Figure 1. Immunohistochemistry was carried out to investigate the expression of CD147 protein in glioma tissues. Sections were incubated overnight with polyclonal antibody against CD147 (1:150; MAB972-100, R@D) at 4 ℃. All slides were counterstained with hematoxylin. Results were graded for staining intensity (0, negative; 1, weak; 2, moderate; 3, strong) and percentage of positive cells,a scale of 0 (negative, <5%), 1+(weak, 6–25%), 2+ (intermediate, 26%-50%), 3+ (intensive>51%) was applied, where the percentage indicates the portion of tumor cells stained.The intensity and proportion scores were added to produce the final score: high(score≥3) and low or none (score = 0-2). Immunohistochemistry analysis revealed that the vast majority of 78 paraffin-embedded archival glioma specimens tested displayed positive CD47 expression, The positive CD147 expression rate was 60.25% (47/78) in glioma tissue and 46.37% exhibited high-level CD147 expression.CD147 had stronger staining in high-grade glioma tissues than low grade glioma tissues (supplementary 1A). As far as clinicopathological characteristics were considered,WHO grade was proved to be associated with overall survival since patients with high grade glioma tend to have poorer overall survival and higher risk of death compared with those of low grade. Patients with low CD147 expression had higher overall survival in high-grade gliomas than those with high CD147 expression (supplementary 1B). We analysed CD147(BSG), its transcriptome in data from 603 glioma patients using the Chinese Glioma Genome Atlas (CGGA) was collected and identified, database (http://www.cgga.org.cn/download_other.jsp).CD147 expression of high-grade glioma(HGG,WHO tumor grade III and IV) was significantly upregulated compared to that of matched low-grade glioma (LGG,WHO tumor grade I and II) tissues (supplementary 1C). High-grade glioma patients with high levels of CD147 exhibited poorer overall survival rate compared with those with low CD147 levels.(supplementary D).

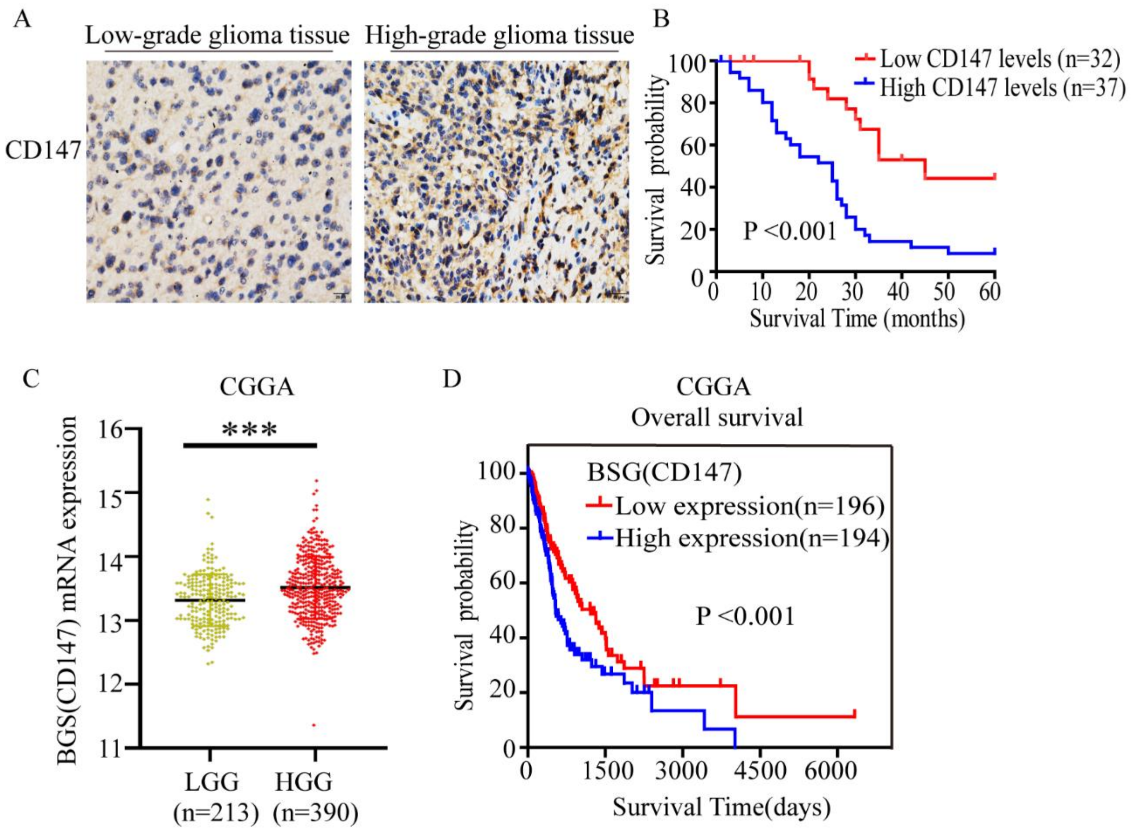

FIGURE 1 Upregulation of CD147 in glioma tissues correlates inversely with the clinical outcome. **(A)** Representative images from IHC assays of paraffin-embedded specimens of a total of 78 primary glioma tissue specimens (magnification X400). CD147 shows stronger staining in hig-grade gliomas tissues (HGG,WHO Grades III to IV) than in low-grade gliomas tissue(LGG,WHO Grades I to II), scale bar 20μm.**(B)** The survival is significantly different between CD147 high- and low-expressing patients within high-grade glioma.The cumulative 5-year survival rate is 41% in the low CD147-expression group (n= 32), compared with 25 % in the high CD-expression group (n=37).**(C)** The levels of CD147 was distinctly upregulated in high grade glioma tissues by analyzing the CGGA datasets(n=603).**(D)** Kaplan–Meier curve indicated higher CD147 expression was unfavorable for patient survival based on the CGGA database. ***P < 0.001.
